# Responses of Intestinal Microbiota and Immunity to Increasing Dietary Levels of Iron Using a Piglet Model

**DOI:** 10.3389/fcell.2020.603392

**Published:** 2020-12-17

**Authors:** Shuai Chen, Xin Wu, Xia Wang, Yirui Shao, Qiang Tu, Huansheng Yang, Jie Yin, Yulong Yin

**Affiliations:** ^1^National Engineering Laboratory for Pollution Control and Waste Utilization in Livestock and Poultry Production, Key Laboratory of Agro-Ecology, Institute of Subtropical Agriculture, Chinese Academy of Sciences, Changsha, China; ^2^University of the Chinese Academy of Sciences, Beijing, China; ^3^College of Animal Science and Technology, Hunan Agriculture University, Hunan Co-Innovation Center of Animal Production Safety, Changsha, China; ^4^Animal Nutrition and Human Health Laboratory, School of Life Sciences, Hunan Normal University, Changsha, China; ^5^Yiyang Vocational Technical College, Yiyang, China; ^6^State Key Laboratory of Microbial Technology, Shandong University, Qingdao, China

**Keywords:** iron overload, pig, diarrhea, immunity, microbiota, *Lactobacillus*

## Abstract

Iron is an essential metal for both animals and microbiota. In general, neonates and infants of humans and animals are at the risk of iron insufficiency. However, excess dietary iron usually causes negative impacts on the host and microbiota. This study aimed to investigate overloaded dietary iron supplementation on growth performance, the distribution pattern of iron in the gut lumen and the host, intestinal microbiota, and intestine transcript profile of piglets. Sixty healthy weaning piglets were randomly assigned to six groups: fed on diets supplemented with ferrous sulfate monohydrate at the dose of 50 ppm (Fe50 group), 100 ppm (Fe100 group), 200 ppm (Fe200 group), 500 ppm (Fe500 group), and 800 ppm (Fe800), separately, for 3 weeks. The results indicated that increasing iron had no significant effects on growth performance, but increased diarrheal risk and iron deposition in intestinal digesta, tissues of intestine and liver, and serum. High iron also reduced serum iron-binding capacity, apolipoprotein, and immunoglobin A. The RNA-sequencing analysis revealed that iron changed colonic transcript profile, such as interferon gamma-signal transducer and activator of transcription two-based anti-infection gene network. Increasing iron also shifted colonic and cecal microbiota, such as reducing alpha diversity and the relative abundance of *Clostridiales* and *Lactobacillus reuteri* and increasing the relative abundance of *Lactobacillus* and *Lactobacillus amylovorus*. Collectively, this study demonstrated that high dietary iron increased diarrheal incidence, changed intestinal immune response-associated gene expression, and shifted gut microbiota. The results would enhance our knowledge of iron effects on the gut and microbiome in piglets and further contribute to understanding these aspects in humans.

## Introduction

Iron is an essential metal for humans and animals, requiring an iron-containing bioactive such as heme protein, enzyme, and iron–sulfur cluster proteins, to maintain essential functions such as sensing, storing, and transporting oxygen, energy metabolism, DNA synthesis, intermediate metabolism and detoxification, and host defense. Iron inadequacy usually causes IDA, and iron deficiency in early life would lead to growth retardation and cognitive disorder (Hershko and Camaschella, 2014). Iron overload, commonly due to the genetic disorder, would induce iron accumulation, contributing to hemochromatosis, cancer, cardiovascular disease, metabolism syndrome, and neurodegenerative disease (Lonnerdal, 2017). Iron is also an indispensable growth factor for microbiota, which complicates with the host to acquire iron for survival (Barton and Acton, 2019). Thus, it is crucial to maintain iron homeostasis, dependent on iron supplementation, absorption, cycling, storage, and interaction between the host and microbiota.

Neonates and infants of humans and animals, such as piglets, are commonly at the risk of iron insufficiency because of high iron requirements, low iron stores, and inadequate dietary supplementation or poor absorptive efficiency. However, excess iron accumulation causes oxidative pressure and further damage to DNA and proteins and peroxide lipids. Prolonged iron supplementation also causes gastroenteric disorder and intestinal microbiota dyshomeostasis and would increase the risk of numerous diseases. Increased body iron stores were recognized as a feature of metabolic syndrome, and high dietary iron levels increased blood glucose levels but decreased HDL cholesterol levels. Excess dietary iron in the high-fat diet increased glucose, insulin, insulin resistance, and liver fat deposition (Choi et al., 2013). Moreover, iron-rich diets, such as red meat, promote the risk of colorectal cancer. In a randomized controlled trial, iron-containing MNP supplementation to 6-month infants for 4 months increased intestinal inflammation and gut pathogen abundance, such as pathogenic *Escherichia coli (E. coli)*, and iron at a dose of 12.5 mg/day in MNPs increased diarrhea risk than that of 2.5 mg/day (Jaeggi et al., 2015). The following controlled intervention trial revealed that MNPs given to 8- to 10-month infants interfered with antibiotic efficiency and promoted the risk of diarrhea (Paganini et al., 2019). As an excellent model for human studies of nutrition, metabolism, neurodevelopment, gut, and microbiota, pigs share many physiological structures and function with humans (Roura et al., 2016; Mudd and Dilger, 2017; Kleinert et al., 2018). Like humans, piglets born with limited iron stores, which will be deficient without exogenous sources, and weaning piglets also suffer from anemic and iron deficiency (Council, 2012; Perri et al., 2016). The dietary iron requirement for post-weaning piglets is recommended at a dose of 80 mg/kg dry matter; however, the dietary iron in commercial feed usually exceed the suggested dose up to three times (Flohr et al., 2016). High dietary iron impairs the gut by increasing intestinal permeability, malondialdehyde abundance, neutrophil infiltration, diarrheal incidence, and reducing villus height of duodenum in weanling pigs; excess iron also disrupts intestinal microbiota, such as enriching coliform bacillus and reducing *Bifidobacterium* spp. in weanling pigs (Lee et al., 2008; Li et al., 2016b). Furthermore, iron uptake is essential to maintain the virulence of pathogens; thus, sequestration of iron is a vital strategy of the host to infection. However, excess iron deposition in the gut bins host’s iron sequestration strategy, leading to pathogen burst to increase the risk of infection and inflammation (Nairz and Weiss, 2020). For example, dietary iron aggravates dextran sulfate sodium-induced colonic inflammation and activates IL-6/IL-11-Stat3 signaling-induced colonic cancer development in mice (Carrier et al., 2006; Chua et al., 2013). Although these studies demonstrated the adverse effects of iron overload on the metabolism of glucose and lipid metabolism, inflammation, and microbiota, few studies revealed the deposition of dietary iron in different intestinal anatomical regions and the region-specific response of the microbiota and intestine.

This study aimed to analyze the effect of dietary gradient iron supplementation on the distribution pattern of iron in the gut lumen and body, intestinal microbiota, and intestine gene expression profile. The results would enhance our knowledge of iron distribution and its effects on the gut and microbiome in piglets and further contributes to the understanding these aspects in humans.

## Materials and Methods

### Animals and Experiment Design

**FIGURE 1:**
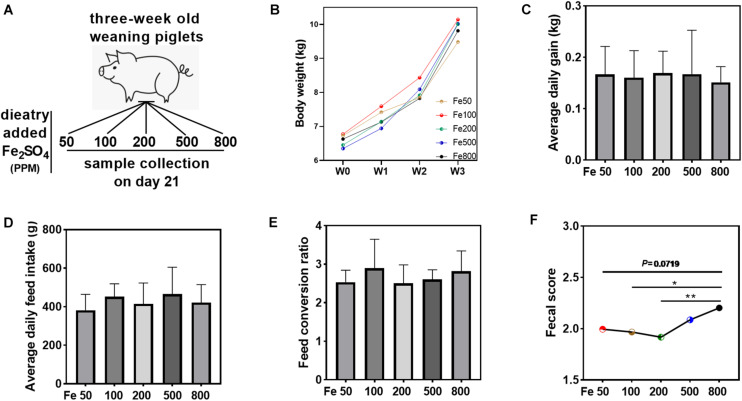
Dietary iron had no significant effects on the growth performance but increased fecal consistency score. Growth performance and fecal consistency scores were evaluated in each group. Schematic showing dietary iron treatment of each group **(A)**. Body weight **(B)**, average daily gain **(C)**, average daily feed intake **(D)**, and feed conversion ratio **(E)** were similar among the five groups. The average fecal score of the 21 days **(F)** in the Fe800 group was also higher than that in the other groups. Ordinary one-way ANOVA or Kruskal–Wallis test was used for the statistical analysis among different groups. Data were presented as mean ± SD. ^∗^*P* < 0.05, ^∗∗^*P* < 0.01.

Sixty healthy weaning piglets (Duroc × Landrace × Landrace, 21 days old, average body weight 6.58 kg), purchased from a commensal farm (Hunan New Wellful, Co., Ltd., Changsha, China), were randomly assigned to six groups: Fe50, Fe100, Fe200, Fe500, and Fe800 groups (Figure 1A). The piglets in the Fe50 group were fed on a corn and soybean meat-based diet [Supplementary Table 1, a formula modified according to NRC 2012 (Council, 2012)], according to our previous study (Zhou et al., 2017), supplemented with 50 ppm ferrous sulfate, while the diets of piglets in Fe100, Fe200, Fe500, and Fe800 were fed our formula supplemented with 100, 200, 500, and 800 ppm ferrous sulfate monohydrate, separately. The piglets were housed individually in an environmentally controlled facility with hard plastic slatted flooring and maintained at an ambient temperature of 25 ± 2°C with free access to diets and drinking water. This experiment was last for 21 days. The body weight, feed intake, and fecal score were detected weekly, and the feed conversation ratio was calculated. The fecal consistency score was assessed by a modified method according to our previous study (Wang et al., 2019): 1 = solid, 2 = pasty, and 3 = liquid. The piglets were sacrificed on day 21 to collect blood, liver tissue, intestinal tissue (including duodenum, jejunum, ileum, and colon), and digesta of cecum and colon. The blood was centrifuged at 3,000 rpm for 10 min for serum separation. All the samples were snap-frozen in liquid nitrogen and stored at −80°C before further processing. The Animal Welfare Committee of the Institute of Subtropical Agriculture, Chinese Academy of Sciences, approved all the animal experimental procedures.

### Iron Status Analysis

Iron levels in duodenal, jejunal, ileal, colonic, liver tissues, and the digesta of jejunum, ileum, cecum, colon, and feces were determined by inductively coupled plasma optical emission spectrometry (ICP-OES; ICP 720 ES; Agilent, United States) according to our previous study (Zhang et al., 2017). The intestinal digesta was freeze dried before assessed. Briefly, the samples were weighed in triplicate, subjected to acid digestion, dried at 260°C, and redissolved in 5 ml of 1% HNO_3_. Then, the samples were diluted with 1% HNO_3_ and subjected to ICP analyses after dilution.

### Conventional Biochemical Measurements

Blood hemoglobin level was tested by the BeneCheck Hemoglobin Test System (BeneCheck, Taiwan, China). Several blood parameters related to the glucose, lipid, and iron metabolism and liver and kidney function were analyzed by Cobas c 311 analyzers (Roche Analytic Instruments, Nutley, NJ, USA.) according to the manufacturer’s instructions. The detected blood parameters were as follows: iron, calcium, magnesium, unsaturated iron-binding capacity, transferrin, soluble transferrin receptors, ceruloplasmin, glucose, total bile acids, triglyceride, cholesterol, HDL, LDL, apolipoprotein, lipoprotein, total protein, alanine aminotransferase, aspartate aminotransferase, albumin, immunoglobin A, IgM, IgG, ureal, uric acid, and creatinine.

### 16S rDNA Sequencing Analysis of Cecal and Colonic Microbiota

16S rDNA sequencing of cecal and colonic microbiota was used for intestinal microbiota analysis according to our previous study (Wu et al., 2020). Briefly, the DNA was extracted from cecal and colonic digesta by Qiagen QIAamp DNA Stool Mini kit (Qiagen, Hilden, Germany) according to the manufacturer’s recommendation. The V3 to V4 regions of bacterial 16S rDNA were selected to analyze using Illumina MiSeq sequencing. The library was generated and sequenced to produce 400 base pair/600 base pair single-end reads. Single-end reads were assigned to the samples based on their unique barcode, and then their barcode and the primer sequence were removed. Sequence analysis was following quality filtering. The sequences with more than 97% similarity were clustered to the same OTU by UPARSE software (v7.0.1001^1^). RDP Classifier (V2.2, Michigan State University Board of Trustees, East Lansing, MI, United States) was used for species annotations, based on the GreenGene database^2^. The MUSCLE software (Version 3.8.31) was used to analyze the phylogenetic relationships of different OTUs to reveal differences among samples and groups and for multiple-sequence alignments. The OTUs were normalized for subsequent analysis of the alpha diversity, beta diversity, and the environmental-factor correlation analysis. The 16S rDNA sequencing and data analysis were performed by a commercial company (Novogene, Co., Ltd., Beijing, China).

### RNA-Seq-Based Reference Transcriptome Analysis of Colonic Tissue

RNA isolation was processed according to our previous study (Chen et al., 2014). Briefly, total RNA was isolated from the liquid nitrogen-frozen colon by TRIZOL regents (Invitrogen, United States) and then treated with DNase I (Invitrogen, United States) according to the manufacturer’s instructions. After quantification and qualification, the RNA was used to generate sequencing libraries by NEBNext^®^ UltraTM RNA Library Prep Kit for Illumina^®^ (NEB, United States) according to the manufacturer’s protocol and then index coded. The index-coded samples were clustered using TruSeq PE Cluster Kit v3-cBot-HS (Illumia) on a cBot Cluster Generation System following the manufacturer’s recommendation and then sequenced on an Illumina Novaseq platform. One hundred fifty base pair paired-end reads were generated for the following data analysis. Hisat2 (v2.0.5) was used for read mapping to the reference genome. FPKM of each gene was calculated after counting the read numbers of each mapped gene using feature Counts (v1.5.0-p3). After gene expression level quantification, DEG analysis was performed by the DESeq2 R package (1.16.1), and *P*-value < 0.05 was assigned as differential expression. The clusterProfiler R package was used to implement Gene Ontology (GO) enrichment analysis and KEGG enrichment analysis of DEGs. PPI analysis of DEGs was based on the STRING database and further analyzed and visualized by Cytoscape (v3.8.0). The whole transcriptome sequencing and data analysis were performed by a commercial company (Novogene, Co., Ltd., Beijing, China).

### Statistical Analyses

The data were preprocessed with Excel 2019 (Microsoft, Redmond, United States). Word 2019 software (Microsoft, Redmond, United States) was used to prepare tables, and GraphPad Prism 8.0 (GraphPad Software, Inc., La Jolla, CA, United States) was used in statistical analysis and figure generation. Ordinary one-way ANOVA or Kruskal–Wallis test was used for the statistical analysis among different groups. Unpaired *t*-test was used to statistically analyze between two groups if the data followed a normal distribution; otherwise, the Wilcoxon signed-rank test was used for the analysis. The results were presented as mean ± standard deviation (SD) or mean ± standard error of the mean (SEM), and *P*-value < 0.05 was considered as statistically significant.

## Results

### Dietary Iron Had No Significant Effects on the Growth Performance but Increased Fecal Consistency Score

Growth performance and fecal consistency scores were evaluated to assess the effects of iron on the growth and diarrheal incidence of the piglets. The results indicated that growth parameters, including body weight, average daily gain, average daily feed intake, and feed conversion ratio, were similar among the five groups (Figures 1B–E). The daily fecal score was shown in Supplementary Figure 1A, and the fecal score on day 21 was significantly higher in the Fe800 group than in other groups (Supplementary Figure 1B). Meanwhile, the average fecal score on the 21 days in the Fe500 group was also higher than in the other groups (Figure 1F).

### Dietary Iron Increased Iron Deposition in Intestinal Digesta, Intestine, Liver, and Serum

**FIGURE 2:**
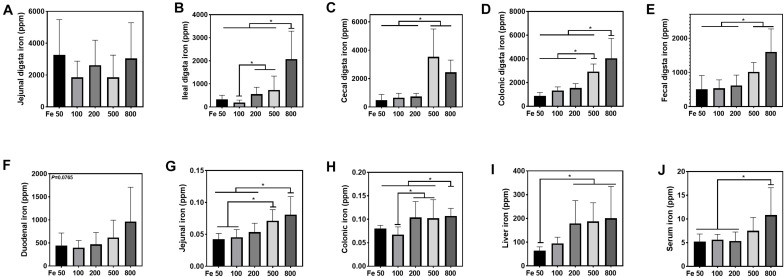
Dietary iron increased iron deposition in intestinal digesta, intestine, liver, and serum. The iron concentration in the jejunal digesta **(A)** was similar among different groups. Iron levels of ileal **(B)**, cecal **(C)**, and colonic digesta **(D)** and feces **(E)** in the Fe800 group were significantly higher than those in the Fe50, Fe100, and Fe200 groups, and iron abundance of cecal and colonic digesta and feces in the Fe500 group was also higher compared with that in the Fe50, Fe100, and Fe200 group. Tissue iron content in the duodenum **(F)**, jejunum **(G)**, colon **(H)**, liver **(I)**, and serum **(J)** went up from Fe50 to Fe800. Ordinary one-way ANOVA or Kruskal–Wallis test was used for the statistical analysis among different groups. Data were presented as mean ± SD. *P < 0.05.

The iron concentration in the jejunal digesta was similar among different groups (Figure 2A). Iron levels of ileal, cecal, and colonic digesta and feces in the Fe800 group was significantly higher than in the Fe50, Fe100, Fe200 groups, and iron abundance of cecal and colonic digesta and feces in the Fe500 group was also higher compared to Fe50, Fe100, and Fe200 groups (Figures 2B–E). The iron content in duodenum, jejunum, colon, liver, and serum went up from the Fe50 to Fe800 group (Figures 2F,J), but not in the ileum (data not shown). Jejunal iron in the Fe800 and Fe500 groups was significantly higher than those of the other three groups and also higher in the Fe500 group than in the Fe50 and Fe100 groups (Figure 2G). Colonic iron in the Fe800 group was higher than that of the other four groups (Figure 2H). Liver iron in the Fe800, Fe500, and Fe200 groups was significantly increased than in the Fe50 group (Figure 2I). Serum iron in the Fe800 group was much higher than in the Fe50–200 groups (Figure 2J).

### Dietary Iron Downregulated Serum Unsaturated Iron-Binding Capacity, Apolipoprotein, and Immunoglobin A

**FIGURE 3:**
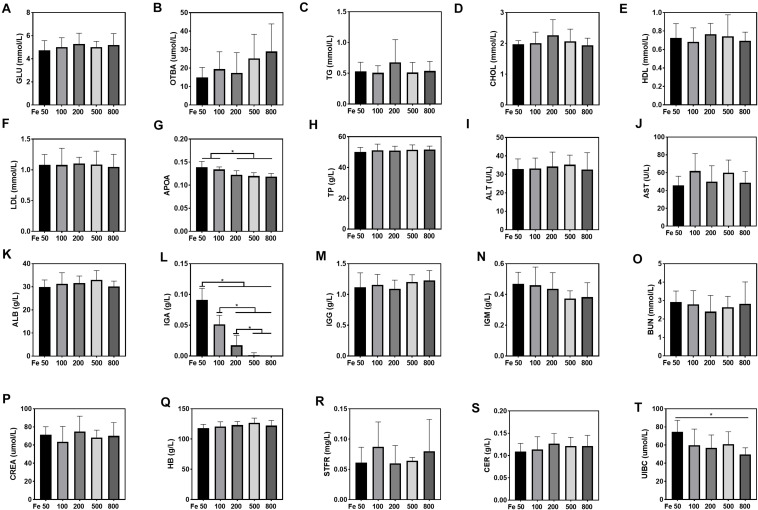
Dietary iron downregulated serum unsaturated iron-binding capacity (UIBC), apolipoprotein (APOA), and immunoglobin A (IGA). Serum glucose (GLU), total bile acids (OTBAs), triglyceride (TG), CHOL, high-density lipoprotein (HDL), low- density lipoprotein (LDL), total protein (TP), alanine aminotransferase (ALT), aspartate amino transferase (AST), albumin (ALB), IgG, and IgM were the same among all the groups **(A–F,H–K,M,N)**. Serum APOA **(G)** and IgA **(L)** were downregulated with dietary iron increasing. Blood HB and serum soluble transferrin receptor (STFR) and ceruloplasmin (CER) were similar among all the groups **(Q–S)**. Serum UIBC **(T)** went down from the Fe50 to Fe800 groups, and UIBC in the Fe50 group was higher than in the Fe800 group. Serum BUN **(O)** and CREA **(P)** were also similar among all the groups. Ordinary one-way ANOVA or Kruskal–Wallis test was used for the statistical analysis among different groups. Data were presented as mean ± SD. *P < 0.05.

Serum UIBC went down in the Fe50 to Fe800 group, and UIBC in the Fe50 group was higher than in the Fe800 group (Figure 3T). HB, STFR, and CER were similar among all the groups (Figures 3Q–S). Serum GLU, OTBA, TG, CHOL, HDL, LDL, TP, ALT, AST, ALB, IgG, and IgM were the same among all the groups (Figures 3A–F,H–K,M,N), but APOA (Figure 3G) and IgA (Figure 3L) were downregulated with dietary iron increasing. Kidney function-related serum BUN and CREA were also similar among all the groups (Figures 3O,P). TSFR and LPA were not detectable in this study.

### Dietary Iron Shifted Gut Microbiology

**FIGURE 4:**
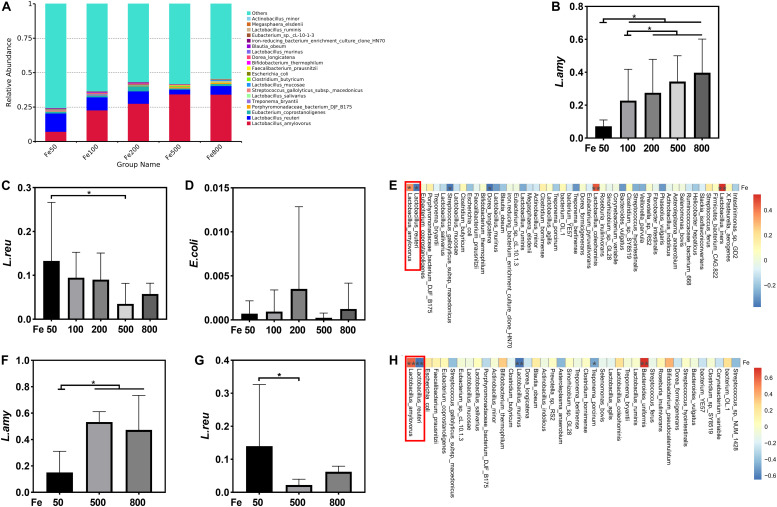
Dietary iron shifted gut microbiology. The relative abundance of *Lactobacillus amylovorus* (*L.amy)* and *Lactobacillus reuteri* (*L.reu)* were dominant in the colon at the species level **(A)**. *L.amy* was significantly higher in the other groups than in the Fe50 group and was more enriched in the Fe200, Fe500, and Fe800 groups than that of the Fe100 group **(B)**. *L.reu* was significantly augmented in the Fe500 group than in the Fe50 group **(C)**, and *Escherichia coli* was not changed among the five groups **(D)**. Correlation analysis indicated that the abundance of *L.amy*, *Lactobacillus coleohominis*, and *Lactobacillus iners* were positively correlated with the iron concentration of colonic digesta, while the abundance of *L.reu*, *Streptococcus gallolyticus*, and *Dorea longicatena* were negatively correlated with the colonic digesta iron concentration **(E)**. Compared with that in the Fe50 group, *L.amy* was higher in the Fe500 and Fe800 groups **(F)**, and *L.reu* was lower in the Fe500 group **(G)**. Correlation analysis also demonstrated that cecal digesta iron positively and negatively correlated with *L.amy* and *L.reu*, separately **(H)**. Ordinary one-way ANOVA or Kruskal–Wallis test was used for the statistical analysis among different groups. Data were presented as mean ± SD. *P < 0.05.

The data, including Raw reads, Clean Reads, Base, AvgLen, Q20, GC%, and Effective%, were generated from 16S RNA sequencing of 38 colonic digesta samples (Supplementary Table 2) for following OUT clustering and taxonomy annotation analysis. 1464 OTUs were detected, and 640 OTUs were the core OUTs shared among the five groups, while 96, 111, 64, 33, and 37 OTUs were specifically enriched in the Fe50, Fe100, Fe200, Fe500, and Fe800 groups, individually (Supplementary Figure 2A). The rarefaction curve (Supplementary Figure 2B) and Good’s-coverage (Table 1) indicated that the sequencing data met the demand for further analysis. Alpha diversity indexes, including Chao, ACE, Shannon, and Simpson, were decreased with increasing iron supplementation, revealing that both microbial community richness and diversity were reduced by high dietary iron (Table 1). The top 10 taxa at each level of colonic microbiota were listed in Table 2. The relative abundances of Bacilli (class level), Lactobacillales (order level), Lactobacillaceae (family level), and *Lactobacillus* (genus level) were upregulated with dietary iron increasing, while Clostridia (class level), Clostridiales (order level), Ruminococcaceae, Lachnospiraceae, Christensenellaceae (family level), and *Ruminococcaceae_UCG-005* (genus level) were downregulated by dietary iron increasing. In the species level, the relative abundance of colonic *Lactobacillus amylovorus (L.amy)*, and *Lactobacillus reuteri (L.reu)* were dominant (Figure 4A). The relative abundance of *L.amy* went down with iron supplementation increasing, while *L.reu* went up (Figures 4B,C). *L.amy* was significantly higher in the other groups than in the Fe50 group and was more enriched in the Fe200, Fe500, and Fe800 groups than that in the Fe100 group (Figure 4B). *L.reu* was significantly augmented in the Fe500 group than in the Fe50 group (Figure 4C), and *E. coli* was not changed among the five groups (Figure 4D). Correlation analysis indicated that the abundance of *L.amy*, *Lactobacillus coleohominis*, and *Lactobacillus iners* were positively correlated with the iron concentration of colonic digesta, while the abundance of *L.reu*, *Streptococcus gallolyticus*, and *Dorea longicatena* were negatively correlated with the colonic digesta iron concentration (Figure 4E). The cecal microbiota in the Fe50, Fe500, and Fe800 groups was also analyzed to support the findings from colonic microbiota analysis, and the changes in microbiota of the cecum was similar with the results from colonic microbiota. Compared with those in the Fe50 group, alpha diversity indexes were significantly decreased in the Fe500 and Fe800 groups, and *Lactobacillus* was also dominant and higher in the Fe800 group than in the Fe50 group (Supplementary Table 4, Supplementary Figure 2C). Compared with those in the Fe50 group, *L.amy* was higher in the Fe500 and Fe800 groups, and *L.reu* was lower in the Fe500 group (Figures 4F,G). And *E. coli* was also similar among the three groups (Supplementary Figure 2D). Correlation analysis also demonstrated that iron concentration in cecal digesta positively and negatively correlated with *L.amy* and *L.reu*, separately (Figure 4H).

**TABLE 1:** Alpha diversity indices of the colonic microbiota of piglets.

	**Fe50**	**Fe100**	**Fe200**	**Fe500**	**Fe800**	**SEM**	***P*-value**
Goods_coverage	0.999	0.999	0.999	0.999	0.999	0.0001	-
Shannon	5.493^a^	5.455^a^	5.000^a^	4.589^ab^	3.983^b^	0.390	0.007
Simpson	0.904^a^	0.887^a^	0.861^ab^	0.823^ab^	0.790^b^	0.0384	0.073
Chao1	513.465^a^	438.437^ab^	443.219^ab^	443.013^ab^	360.0693^b^	39.849	0.056
ACE	523.693^a^	450.541^ab^	487.018^ab^	454.794^ab^	371.591^b^	38.282	0.024

*Goods_coverage, richness estimator (Chao1 and ACE), and diversity estimator (Shannon and Simpson) were analyzed by mothur software of the piglets from the Fe50 (*n* = 8), Fe100 (*n* = 8), Fe200 (*n* = 8), Fe500 (*n* = 6), and Fe800 (*n* = 6) groups. Ordinary one-way ANOVA or Kruskal–Wallis test was used for the statistical analysis among the different groups. Data were presented as mean ± SEM. Means in a row that have no superscript shared are significantly different from each other (*P* < 0.05).*

**TABLE 2:** The top 10 taxa at the phylum, class, order, family, and genus level of colonic microbiota.

**Taxonomy**	**Fe50**	**Fe100**	**Fe200**	**Fe500**	**Fe800**	**SEM**	***P*-value**
**Phylum**							
Firmicutes	0.9001	0.9020	0.9034	0.9127	0.9409	0.0158	0.6079
Bacteroidetes	0.0592	0.0614	0.0571	0.0527	0.0342	0.0128	0.5260
Proteobacteria	0.0024	0.0032	0.0085	0.0015	0.0134	0.0005	0.1113
Spirochaetes	0.0127	0.0114	0.0061	0.0088	0.0029	0.0012	0.4731
Actinobacteria	0.0040	0.0072	0.0065	0.0053	0.0015	0.0003	0.4461
Tenericutes	0.0143	0.0078	0.0148	0.0146	0.0044	0.0018	0.1412
Cyanobacteria	0.0043	0.0059	0.0025	0.0018	0.0011	0.0006	0.3213
Saccharibacteria	0.0021	0.0009	0.0007	0.0023	0.0016	0.0002	0.5717
Verrucomicrobia	0.0005	0.0000	0.0001	0.0000	0.0000	0.0000	0.2362
Chlamydiae	0.0000	0.0000	0.0000	0.0000	0.0000	0.0000	0.9279
**Class**							
Bacilli	0.3610^c^	0.4572^b^	0.4856^b^	0.4452^b^	0.7503^a^	0.0413	0.0031
Clostridia	0.5104^a^	0.3989^b^	0.3729^b^	0.4374^b^	0.1780^c^	0.0332	0.0010
Erysipelotrichia	0.0268	0.0427	0.0409	0.0284	0.0122	0.0017	0.7686
Bacteroidia	0.0591	0.0612	0.0571	0.0527	0.0342	0.0128	0.7187
Gammaproteobacteria	0.0014	0.0022	0.0063	0.0013	0.0127	0.0006	0.1493
Epsilonproteobacteria	0.0007	0.0007	0.0015	0.0000	0.0006	0.0000	0.6812
Unidentified_Spirochaetes	0.0127	0.0114	0.0061	0.0088	0.0029	0.0012	0.4731
Mollicutes	0.0143	0.0078	0.0148	0.0146	0.0044	0.0018	0.1412
Unidentified_Actinobacteria	0.0002	0.0019	0.0029	0.0024	0.0004	0.0001	0.6667
Coriobacteriia	0.0038	0.0053	0.0036	0.0029	0.0011	0.0002	0.2964
**Order**							
Lactobacillales	0.361^c^	0.4571^b^	0.4855^b^	0.4452^b^	0.7501^a^	0.0413	0.0032
Clostridiales	0.5100^a^	0.3987^b^	0.3729^b^	0.4374^ab^	0.1779^c^	0.0331	0.0009
Erysipelotrichales	0.0268	0.0427	0.0409	0.0284	0.0122	0.0017	0.5534
Bacteroidales	0.0591	0.0612	0.0571	0.0527	0.0342	0.0128	0.7187
Enterobacteriales	0.0008	0.0013	0.0053	0.0004	0.0085	0.0003	0.4038
Campylobacterales	0.0007	0.0007	0.0015	0.0000	0.0006	0.0000	0.6812
Spirochaetales	0.0127	0.0114	0.0061	0.0088	0.0029	0.0012	0.4731
Mollicutes_RF9	0.0142	0.0077	0.0148	0.0146	0.0043	0.0018	0.1406
Coriobacteriales	0.0038	0.0053	0.0036	0.0029	0.0011	0.0002	0.2964
Pasteurellales	0.0005	0.0006	0.0008	0.0009	0.0041	0.0003	0.8905
**Family**							
Lactobacillaceae	0.3521^c^	0.4554^b^	0.4834^b^	0.4422^b^	0.7451^a^	0.0428	0.0033
Clostridiaceae_1	0.0923	0.0270	0.0196	0.0904	0.0094	0.0039	0.2508
Ruminococcaceae	0.2544^a^	0.2635^a^	0.2479^a^	0.2353^a^	0.121^b^	0.0201	0.0303
Erysipelotrichaceae	0.0268	0.0427	0.0409	0.0284	0.0122	0.0017	0.5534
Peptostreptococcaceae	0.0034	0.0020	0.0014	0.0092	0.0013	0.0006	0.2217
Lachnospiraceae	0.1018^a^	0.0784^ab^	0.0813^ab^	0.0797^ab^	0.0341^b^	0.0052	0.0255
Enterobacteriaceae	0.0008	0.0013	0.0053	0.0004	0.0085	0.0003	0.4038
Christensenellaceae	0.0501^a^	0.0215^ab^	0.0152^ab^	0.0170^ab^	0.0090^b^	0.0039	0.0154
Bacteroidales_S24-7_group	0.0258	0.0297	0.0220	0.0271	0.0181	0.0073	0.9648
Streptococcaceae	0.0088	0.0017	0.0021	0.0029	0.0048	0.0005	0.3046
**Genus**							
*Lactobacillus*	0.3521^c^	0.4554^b^	0.4834^b^	0.4422^b^	0.7451^a^	0.0428	0.0033
Clostridium_sensu_stricto_1	0.0907	0.0265	0.0181	0.0887	0.3768	0.0039	0.2551
*Subdoligranulum*	0.0184	0.0557	0.0393	0.0276	0.0130	0.0038	0.1211
*Romboutsia*	0.0007	0.0002	0.0001	0.0035	0.0091	0.0000	0.3648
*Faecalibacterium*	0.0111	0.0294	0.0275	0.0352	0.0061	0.0028	0.4615
Ruminococcaceae_UCG-014	0.0553	0.0566	0.0506	0.0698	0.0243	0.0105	0.6604
Lachnospiraceae_XPB1014_group	0.0446	0.0125	0.0243	0.0370	0.0232	0.0031	0.2903
*Escherichia–Shigella*	0.0008	0.0013	0.0053	0.0004	0.0089	0.0003	0.4043
Ruminococcaceae_UCG-005	0.0472^a^	0.0188^ab^	0.0146^ab^	0.0168^ab^	0.0088^b^	0.0046	0.0154
*Streptococcus*	0.0088	0.0017	0.0021	0.0029	0.0070	0.0005	0.3034

*The top 10 microbial population at different taxonomy levels in the colon of piglets from the Fe50 (*n* = 8), Fe100 (*n* = 8), Fe200 (*n* = 8), Fe500 (*n* = 6), and Fe800 (*n* = 6) groups. Ordinary one-way ANOVA or Kruskal–Wallis test was used for the statistical analysis among the different groups. Data were presented as mean ± SEM. Means in a row that have no superscript shared are significantly different from each other (*P* < 0.05).*

### Dietary Iron Changed Colonic Transcript Profiles

**FIGURE 5:**
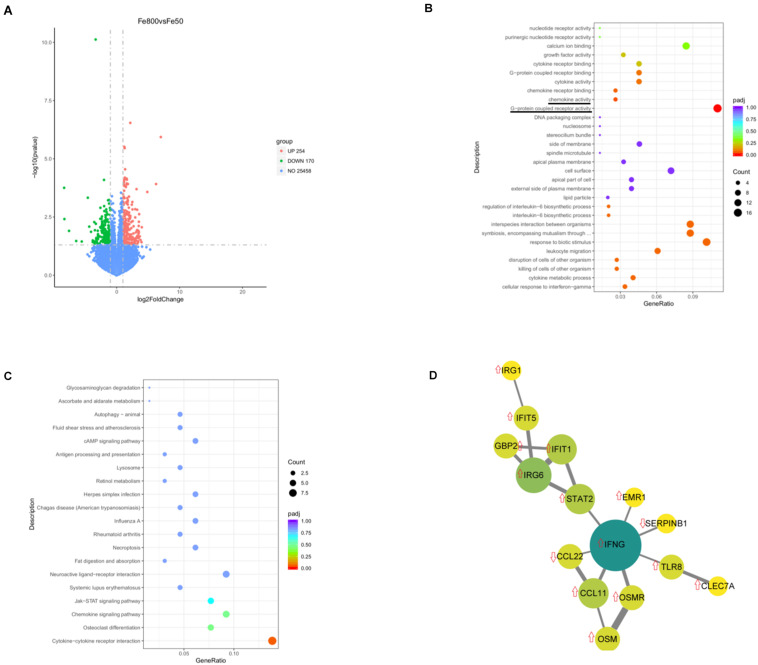
Dietary iron changed colonic transcript profiles. Volcano diagram shows that more than 25,000 gene expressions were detected and quantitated and 424 genes were differential expression genes (DEGs), including 254 upregulated DEGs and 170 downregulated genes **(A)**. Gene Ontology (GO) enrichment analysis showed that G-protein-coupled receptor activity and chemokine activity were significantly enriched **(B)**, while Kyoto Encyclopedia of Genes and Genomes (KEGGs) enrichment analysis indicated that the cytokine–cytokine receptor interaction pathway was significantly enriched **(C)**. The largest connected component of the protein–protein interactions (PPIs) network is shown in **(D)**. Fe50 group (*n* = 5) and Fe800 (*n* = 5) group.

We used an RNA-Seq-based reference transcriptome analysis method to evaluate the effect of dietary iron on colonic gene expression. Data preprocessing statistics and quality control of RNA-sequencing were shown in Supplementary Table 5. More than 25,000 gene expressions were detected and quantitated, and 424 genes were DEGs, including 254 upregulated and 170 downregulated (Figure 5A). The top 20 up/downregulated genes, including IFIT1, ITGBL1, IFNG, TLR8, IFIT5, SERPINB1, and CCL22, were presented in Table 3. GO enrichment analysis showed that G-protein-coupled receptor activity and chemokine activity were significantly enriched (Figure 5B), while KEGG enrichment analysis indicated that the cytokine–cytokine receptor interaction pathway was significantly enriched (Figure 5C). The largest connected component of the PPI network, as shown in Figure 5D, was related to immunity and inflammation.

**TABLE 3:** The top 20 up/downregulatory colonic gene expressions.

**gene_name**	**Fe800**	**Fe50**	**log2FoldChange**	***P*-value**
**Upregulated**				
NEURL3	254.0	56.6	2.2	2.86354E-07
PTPRO	106.3	43.8	1.3	3.4817E-06
MS4A7	248.0	97.3	1.3	2.8176E-05
IFIT1	1,451.6	592.2	1.3	6.44434E-05
ITGBL1	250.9	111.0	1.2	8.15475E-05
OSCAR	73.4	32.3	1.2	9.8907E-05
ACOD1	72.6	21.0	1.8	0.0001
OXTR	63.4	0.8	6.3	0.0001
IFNG	57.1	21.2	1.4	0.0001
PON3	17.4	4.4	2.0	0.0004
cC1QA	4,308.3	1,945.4	1.1	0.0004
FBXO39	163.9	79.3	1.0	0.0005
TLR8	363.8	164.3	1.1	0.0005
THRSP	533.7	165.6	1.7	0.0005
MS4A8	159.4	46.3	1.8	0.0006
TOPAZ1	65.7	19.5	1.7	0.0007
P2RY12	53.0	25.3	1.1	0.0008
IFIT5	1,280.4	609.9	1.1	0.0009
ADGRE1	69.6	20.4	1.8	0.0009
S100A12	17.3	2.2	3.0	0.0015
**Downregulated**				
GIF	9.2	93.0	−3.3	7.53398E-11
RPS14	17.8	45.9	−1.4	0.0006
ESPN	38.3	88.6	−1.2	0.0008
NTNG2	10.8	41.9	−2.0	0.0018
SERPINB1	10.2	27.6	−1.4	0.0024
TAPBPL	5.0	17.1	−1.8	0.0032
APELA	1.5	8.0	−2.4	0.0038
CCL22	75.4	206.2	−1.5	0.0040
LAMP3	6.1	20.4	−1.7	0.0051
KCNK10	2.4	10.6	−2.2	0.0066
ACTA1	16.5	101.2	−2.6	0.0086
KCTD16	2.5	12.2	−2.2	0.0097
ANKRD1	2.2	7.6	−1.9	0.0103
ECEL1	2.6	9.5	−1.8	0.0116
LSR	0.2	2.9	−3.5	0.0117
CCDC114	72.4	174.0	−1.3	0.0129
TXK	26.6	59.7	−1.2	0.0148
PYGM	11.5	35.4	−1.6	0.0161
KCNQ1OT1_1	0.4	3.0	−3.0	0.0172
CYP4F22	7.2	23.8	−1.7	0.0185

*The top 20 up/downregulatory differential expression genes (DEGs) of colon from the Fe50 (*n* = 5) and Fe800 (*n* = 5) groups.*

## Discussion

This study explored excess dietary iron on the growth performance and iron deposition in the host and gut lumen, intestine gene expression profile, and intestinal microbiota. The results demonstrated increasing iron supplementation increased diarrheal risk, increased iron deposition (in intestinal digesta, intestine, liver, and serum), and reduced serum UIBC, APOA, and IgA. Excess iron also shifted gut microbiota and colonic gene expression profiles.

Iron is essential for neonates and infants, and adequate iron supplementation contributes to the growth and development in early life, especially in IDA individuals (Black et al., 2004). However, excess iron supplementation is also harmful to host and microbiota (Paganini et al., 2016). O’donovan et al. (1963) reported that a basal diet containing 65 ppm iron with 15 ppm iron supplementation promoted growth rate and feed consumption, and the basal diet supplemented with excess iron, up to 4,000 ppm but not less than 3,000 ppm, reduced the growth rate of pigs. Coincidently, we found that increasing dietary iron supplementation (up to 800 ppm) had no significant effects on growth performance. However, inconsistent results were reported that 4 weeks of dietary supplemented with iron (250 ppm) reduced ADG of piglets, although it did not affect ADFI and ADG to ADFI ratio (Lee et al., 2008). The difference between the results might come from the treatment time or feed formulation. Although it did not affect growth performance, increasing iron increased diarrheal risk; the result was consistent with previous studies in humans and animals (Rincker et al., 2004; Paganini and Zimmermann, 2017). It is still inconclusive of the mechanism of excess iron induced-diarrhea, and the current consensus is that overloaded iron promotes oxidative stress and disrupts intestinal and microbiota function, further leading to diarrhea (Paganini et al., 2016).

Because it excretes very little, iron absorption and metabolism is finely regulated. The bioavailability of iron is limited, ranging from 2–20% for non-heme iron and 15–35% for heme iron, and is inversely related to iron stores (South et al., 2000). In this study, intestinal digesta iron deposition in the highest iron group is up to six times than that in the lowest iron group. Furthermore, the iron content of the high-iron groups excessed up to two times in the intestinal tissue and serum, and three times in the liver. A large amount of iron accumulation would generate free radical species via catalyzing the Fenton reaction, which might be further leading to oxidative stress, inflammation, and microbiota disruption (Nairz and Weiss, 2020). Dietary supplement with 25 g/kg of carbonyl iron induced oxidative stress in the mitochondria, and long-term excess dietary iron damaged liver mitochondrial function (Volani et al., 2017; Atarashi et al., 2018). Serum creatinine and uric acid were increased in iron-overload thalassemia patients, indicating iron overload corrected with kidney dysfunction (Rasool et al., 2016). However, no significant changes were observed in the serum parameter relation with liver and kidney functions in this study. The inconsistent findings may be related to different animal models or iron handing. Increasing iron also did not affect serum Glu and lipid-related metabolites except for APOA, which was coincident with the previous study that iron status was negatively associated with APOA (Zhou et al., 2020).

Previous studies demonstrated that dietary iron (229.2 ± 1.9 μg/g) increased fecal calprotectin, a biomarker of intestinal inflammation, in Kenyan infants (Jaeggi et al., 2015). Dietary iron promoted colonic inflammation and accelerated tumorigenesis through activating the IL-6/IL-11/Stat3 signaling pathway in mice (Chua et al., 2013). Dietary iron (520 mg/kg, Fe_2_SO_4_) promoted duodenal neutrophil counts and inflammation-related genes in pigs (Li et al., 2016a). In this study, RNA-sequencing analysis of the colon revealed that the cytokine–cytokine receptor interaction pathway was enriched. PPI network analysis revealed the relation of many DEGs, such as interferon-gamma (IFN-γ) and signal transducer and activator of transcription 2 (STAT2). IFN-γ is well-recognized for its antivirus role and is also essential in resistance to bacteria infection (Shtrichman and Samuel, 2001). CLEC7A, a member of C-type lectin receptors, is produced by myeloid cells to sense pathogens and bacterial infections and could activate immune cells in diarrhea-predominant irritable bowel syndrome (Chi et al., 2020). CLE7A and toll-like receptors (TLRs) could be independent or together to activate immunocytes, such as dendritic cells and T-helper-type 1 (Th1) cells, further inducing IFN-γ production (Loures et al., 2015; Perkins and Vogel, 2015; Zimara et al., 2018). IFN-induced proteins with tetratricopeptide repeats (IFITs) could be generated through the IFN-γ–JAK–STAT1/2 pathway to fight against pathogen infection, and IFIT1-deficiency mice are more sensitive to *Burkholderia cenocepacia* (Van Treuren and Dodd, 2020; Zheng et al., 2020). Oncostatin M treatment promoted peptide-pulsed HepG2 cell-induced IFN-γ production by cytotoxic T cells (Larrea et al., 2009). Th1-associated chemokines CCL11 and Th17-associated chemokines CCL22 are elevated in the eye and lymph nodes of IFN-γ-deficiency mice (Su et al., 2007). Th1-immnue response is vital to resist intracellular pathogens, and IFN-γ could limit iron export out of enterocyte by inhibiting ferroportin-1 (Cassat and Skaar, 2013). In macrophage, IFN-γ could stimulate hepatic hepcidin expression through STAT1 (Sow et al., 2009). These reports and our data suggested that the changes of IFN-γ-STAT2 based gene network induced by high level of iron might be the response to limit iron absorption or anti-infection. Our results also revealed that increasing iron reduced serum IgA, which was also observed in children with beta-thalassemia, indicating that iron overload might impair humoral immunity (Hagag et al., 2016). Collectively, high dietary iron changed intestinal gene expression profile, especially for immune genes.

A complex microbial community inhabits the intestine, perceives metal ion limitation or excess via metalloregulatory proteins, and acquires and stores metal in starvation or efflux them while in excess, which would induce cell growth arrest or even death (Chandrangsu et al., 2017). The host tightly regulates pathogen iron access to restrict infection by iron regulatory systems such as hepcidin and lactoferrin. The competition of bacteria with the host for iron is essential for the maintenance of indigenous microbial populations and host health (Chandrangsu et al., 2017). Overload oral iron causes excess iron deposition in the intestine and changes intestinal microbiota in humans and animals (Paganini and Zimmermann, 2017; Ding et al., 2020). Some gram-negative bacteria, such as *Salmonella*, *Shigella*, and pathogenic *E. coli*, require iron for virulence and colonization (Kortman et al., 2014). However, some gram-positive bacteria, such as *Lactobacillus acidophilus*, and unclassical bacteria, such as *Borrelia burgdorferi*, do not need iron (Sabine and Vaselekos, 1967; Posey and Gherardini, 2000). A review by Paganini and Zimmermann (2017) demonstrated that both iron fortification and supplementation in infants and children could disrupt intestinal flora by decreasing bifidobacteria and lactobacilli and increasing enterobacteria such as enteropathogenic *E. coli*. Dietary iron supplemented with iron (up to 250 mg/kg) for 2 weeks linearly increased diarrheal incidence and fecal coliform bacteria in piglets, while the total anaerobic bacteria population was reduced, and coliform bacteria was increased, in diarrheal feces compared with those in normal feces (Lee et al., 2008). A recent study also reported that dietary iron, at a dose of 3,000 ppm, but not 300 ppm, for 4 weeks decreased Clostridiales, Faecalibacterium, and Prevotellaceae, and increased *Desulfovibrio* and *Anaerovibrio* in the cecum of piglets, compared with those of controls (Ding et al., 2020). These studies indicate the regulatory role of dietary iron in intestinal bacteria. Our results also indicated that increasing dietary iron changed the intestinal microbiota, such as decreasing community richness and diversity, increasing Lactobacillus and *L.amy*, and reducing Clostridiales and *L.reu*. However, other populations, such as *E. coli* and bifidobacteria, were unchanged. *Lactobacillus* species, such as *L.reu*, was reported as the main sensors to monitor the gut iron level and were robust in response to intestinal iron deficiency and arrest host iron absorption by its metabolites, for example, reuterin-producing *L.reu* inducing the inhibition of HIF-2a gene expression (Das et al., 2020). *L.amy* is isolated from swine and a potential probiotic bacterium that could produce lactate, acetate, amylovorin, and phytase (Moreno et al., 2008). Together, these data suggest that increasing dietary iron could shift intestinal flora, such as reducing alpha diversity and augmenting the relative abundance of *Lactobacillus*. However, how iron deposition affect the absolute abundance of total bacteria and *Lactobacillus* remains unclear that requires further study.

## Conclusion

In summary, this study reveals the effects of increasing dietary iron on the distribution pattern of iron in the host and gut lumen, intestinal microbiota, and intestine gene expression profile. The results demonstrate that high dietary iron increases diarrheal incidence, changes intestinal immune response-associated gene expression, and shifts gut microbiota. Further studies are needed to analyze the interaction of *Lactobacillus* and the host under conditions of iron excess.

## Data Availability Statement

The datasets presented in this study can be found in online repositories. The names of the repository/repositories and accession number(s) can be found in the article/ Supplementary Material.

## Ethics Statement

The animal study was reviewed and approved by the Animal Welfare Committee of the Institute of Subtropical Agriculture, Chinese Academy of Sciences.

## Author Contributions

HY, SC, and YY designed the experiments, which was performed by SC, XWu, and XWa. SC, QT, and JY analyzed the data. JY and YS prepared the tables and figures. SC and XWu prepared the manuscript. HY, QT, and YY revised the manuscript. All the authors reviewed the manuscript.

## Conflict of Interest

The authors declare that the research was conducted in the absence of any commercial or financial relationships that could be construed as a potential conflict of interest.

## Footnotes

1 https://drive5.com/usearch/

2 https://greengenes.secondgenome.com

## Supplementary Material

Supplementary Figure 1 Dietary iron had no significant effects on the growth performance but increased fecal consistency score. The daily fecal score was shown **(A)**. The fecal score on d21 was significantly higher in the Fe800 group than in other groups **(B)**. Ordinary one-way ANOVA or Kruskal–Wallis test was used for the statistical analysis among different groups. ^∗∗^*P* < 0.01 and ^∗∗∗^*P* < 0.001.

Supplementary Figure 2 Dietary iron shifted gut microbiology. Venn diagram showed colonic shared and unique OTUs among different groups **(A)**. The rarefaction curve of colonic microbial in each group **(B)**. The relative abundance of species in the cecum at the species level was shown **(C)**. Cecal *E. coli* was similar among each group **(D)**. Ordinary one-way ANOVA or Kruskal–Wallis test was used for the statistical analysis among different groups. ^∗^*P* < 0.05.

## Abbreviations

ALB: albumin
ALT: alanine aminotransferase
APOA: apolipoprotein
AST: aspartate aminotransferase
BUN: ureal
Ca: calcium
CER: ceruloplasmin
CHHOL: cholesterol
CREA: creatinine
DEG: differential expression gene
*E. coli*: Escherichia coli
GLU: glucose
GO: Gene Ontology
HDL: high-density lipoprotein
IDA: iron-deficiency anemia
IFN- γ: interferon gamma
IgA: immunoglobin A
KEGG: Kyoto Encyclopedia of Genes and Genomes
*L.amy*: *Lactobacillus amylovorus*
*L.reu*: *Lactobacillus reuteri*
LDL: low-density lipoprotein
LPA: lipoprotein
Mg: magnesium
MNPs: iron-containing micronutrient powders
OTBA: total bile acids
OTU: operational taxonomic unit
PPIs: protein–protein interactions
STAT2: signal transducer and activator of transcription 2
STFR: soluble transferrin receptors
TG: triglyceride
Th1: T helper -type 1
TP: total protein
TSFR: transferrin
UA: uric acid
UIBC: unsaturated iron-binding capacity.
