# Supplementary figures and images for: Responses of Intestinal Microbiota and Immunity to Increasing Dietary Levels of Iron Using a Piglet Model
Source: Front Cell Dev Biol. 2020 Dec 17;8:603392. doi: 10.3389/fcell.2020.603392 (PMC7773786; doi:10.3389/fcell.2020.603392)

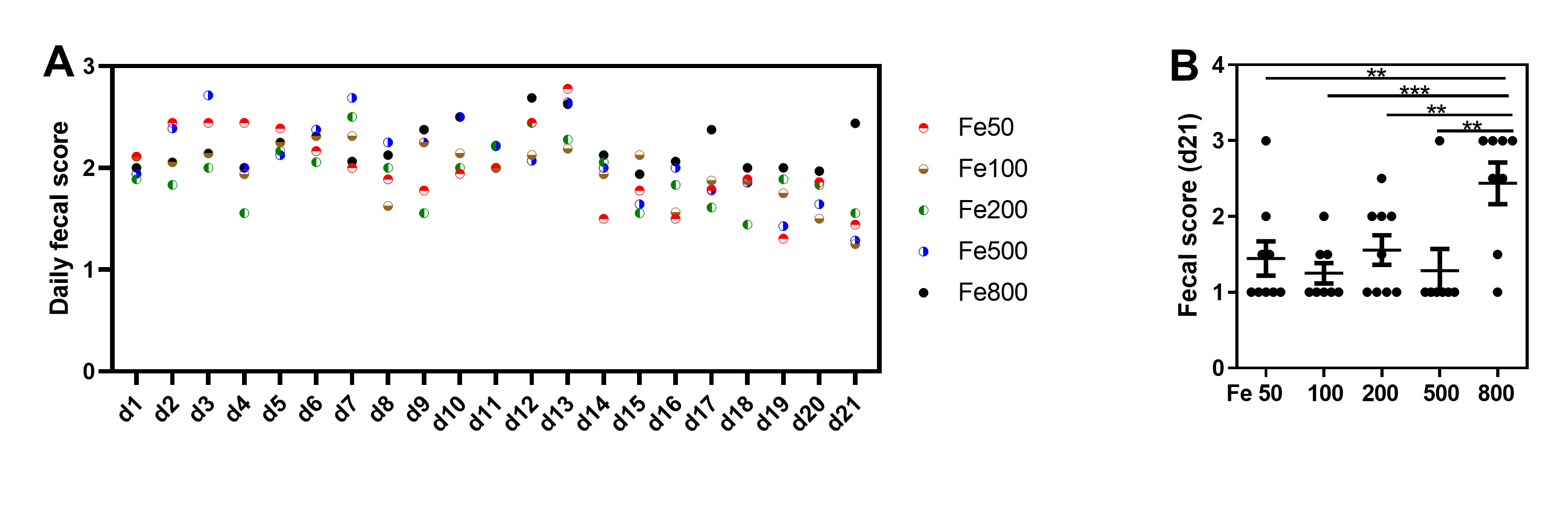

Supplement: Supplementary Figure 1 — Dietary iron had no significant effects on the growth performance but increased fecal consistency score. The daily fecal score was shown (A). The fecal score on d21 was significantly higher in the Fe800 group than in other groups (B). Ordinary one-way ANOVA or Kruskal–Wallis test was used for the statistical analysis among different groups. ∗∗P < 0.01 and ∗∗∗P < 0.001. [file Image_1.TIF]

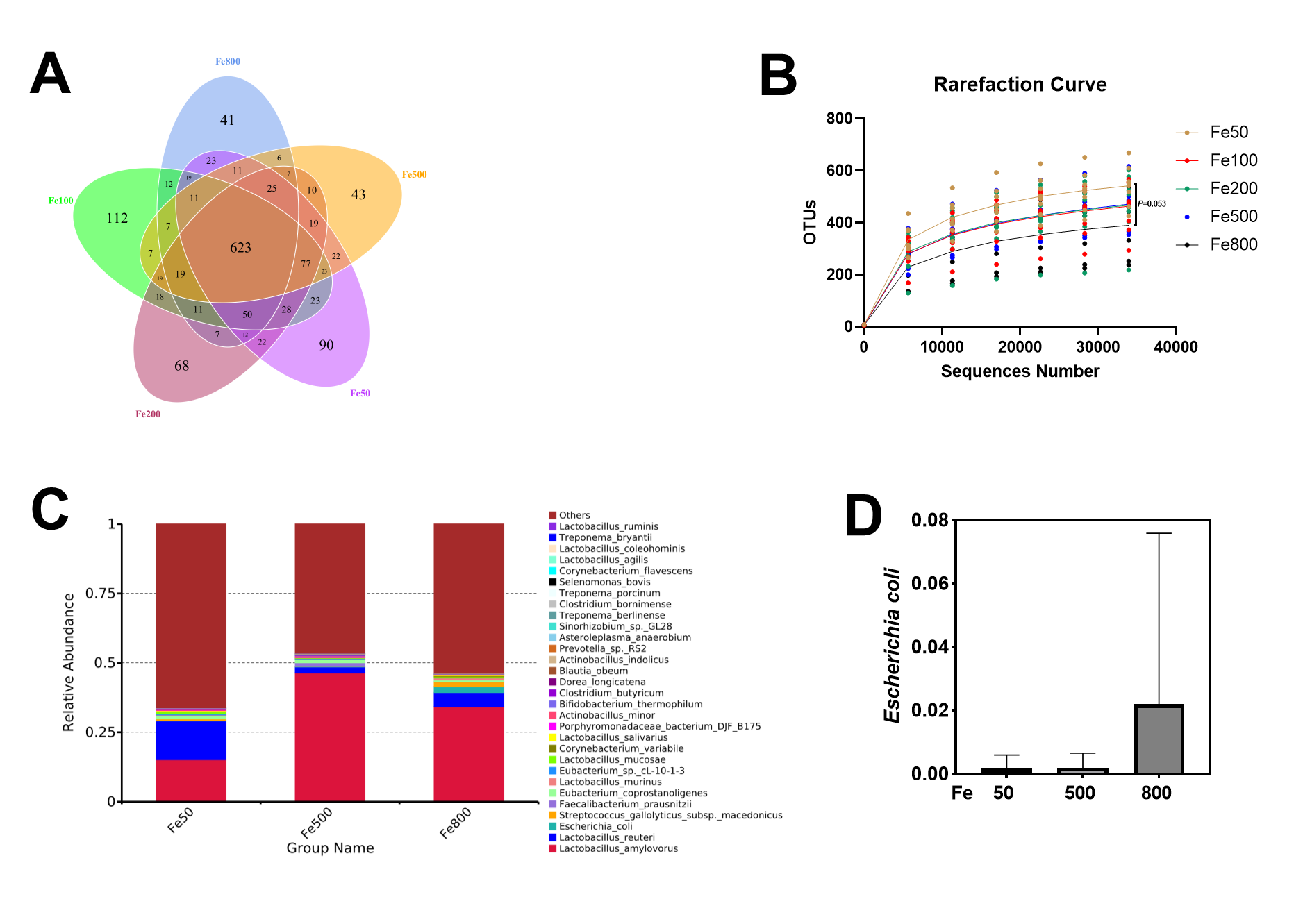

Supplement: Supplementary Figure 2 — Dietary iron shifted gut microbiology. Venn diagram showed colonic shared and unique OTUs among different groups (A). The rarefaction curve of colonic microbial in each group (B). The relative abundance of species in the cecum at the species level was shown (C). Cecal E. coli was similar among each group (D). Ordinary one-way ANOVA or Kruskal–Wallis test was used for the statistical analysis among different groups. ∗P < 0.05. [file Image_2.TIF]
