# Supplementary material for: Responses of Intestinal Microbiota and Immunity to Increasing Dietary Levels of Iron Using a Piglet Model
Source: Front Cell Dev Biol. 2020 Dec 17;8:603392. doi: 10.3389/fcell.2020.603392 (PMC7773786; doi:10.3389/fcell.2020.603392)
Supplement: Supplementary file 3 [file Table_1.DOCX]

**Supplementary Table 1 Formulation of the basal diet**

| **Ingredients, %** | **content** |
| --- | --- |
| Corn | 61 |
| Soybean | 10 |
| Extruded Soybean | 8 |
| Fermented Soybean | 5 |
| Fish meal | 3 |
| Whey powder | 8 |
| Soybean oil | 0.7 |
| Sucrose | 1 |
| Limestone powder | 0.84 |
| Premix | 2.46 |
| Calcium dihydrogen phosphate | 0.78 |
| Salt | 0.37 |
| Lysine HCl (98%) | 0.64 |
| Anti-oxidant | 0.1 |
| minerals | 0.15 |
| Vitamins | 0.04 |
| Zeolite powder | 0.38 |
| **Analyzed chemical composition** |  |
| Dry matter | 86 |
| Crude protein | 18.89 |
| Ether extract | 3.9 |
| DE, MJ/kg | 14.51 |

**Supplementary Table 2 Data pre-processing statistics and quality control of 16sRNA-sequencing**

| **Sample** | **Raw_**  **reads(#)** | **Clean_**  **Reads(#)** | **Base(nt)** | **AvgLen(nt)** | **Q20** | **GC%** | **Effective%** |
| --- | --- | --- | --- | --- | --- | --- | --- |
| Fe50-1 | 52088 | 41857 | 17282886 | 412 | 86.47 | 52.79 | 80.36 |
| Fe50-2 | 65533 | 62573 | 26294776 | 420 | 86.59 | 52.33 | 95.48 |
| Fe50-3 | 69827 | 63050 | 26286745 | 416 | 87.37 | 52.56 | 90.29 |
| Fe50-4 | 62028 | 58363 | 24633491 | 422 | 87.03 | 51.41 | 94.09 |
| Fe50-5 | 64735 | 62639 | 26290120 | 419 | 88.14 | 52.68 | 96.76 |
| Fe50-6 | 58455 | 56023 | 23179937 | 413 | 83.53 | 52.78 | 95.84 |
| Fe50-7 | 59195 | 56450 | 23397812 | 414 | 82.45 | 53.06 | 95.36 |
| Fe50-8 | 67434 | 62422 | 25631865 | 410 | 86.69 | 52.64 | 92.57 |
| Fe100-1 | 69416 | 63906 | 27077226 | 423 | 85.97 | 51.37 | 92.06 |
| Fe100-2 | 98045 | 91492 | 38381531 | 419 | 82.09 | 51.96 | 93.32 |
| Fe100-3 | 63801 | 57643 | 23849123 | 413 | 85.33 | 52.71 | 90.35 |
| Fe100-4 | 75996 | 73202 | 30502019 | 416 | 84.65 | 53.43 | 96.32 |
| Fe100-5 | 82533 | 80141 | 33534448 | 418 | 83.18 | 51.56 | 97.1 |
| Fe100-6 | 60161 | 56314 | 23525474 | 417 | 85.83 | 52.59 | 93.61 |
| Fe100-7 | 62237 | 56096 | 23478282 | 418 | 84.9 | 51.74 | 90.13 |
| Fe100-8 | 88627 | 80248 | 33695004 | 419 | 82.53 | 51.94 | 90.55 |
| Fe200-1 | 77982 | 71934 | 29952223 | 416 | 83.64 | 52.06 | 92.24 |
| Fe200-2 | 63241 | 58799 | 24410832 | 415 | 85.39 | 52.66 | 92.98 |
| Fe200-3 | 56479 | 54234 | 22717432 | 418 | 84.73 | 52.46 | 96.03 |
| Fe200-4 | 66836 | 60959 | 25888697 | 424 | 83.51 | 51.74 | 91.21 |
| Fe200-5 | 77358 | 72906 | 30277298 | 415 | 86.27 | 52.16 | 94.24 |
| Fe200-6 | 87847 | 80204 | 33969584 | 423 | 83.26 | 51.19 | 91.3 |
| Fe200-7 | 87143 | 84371 | 35228786 | 417 | 84.54 | 52.28 | 96.82 |
| Fe200-8 | 93308 | 84637 | 35771484 | 422 | 81.12 | 51.4 | 90.71 |
| Fe500-1 | 84552 | 80133 | 33502180 | 418 | 81.19 | 51.65 | 94.77 |
| Fe500-2 | 100614 | 95491 | 39941049 | 418 | 83.2 | 51.68 | 94.91 |
| Fe500-3 | 82563 | 80242 | 33769169 | 420 | 82.74 | 51.55 | 97.19 |
| Fe500-4 | 73595 | 70142 | 29401094 | 419 | 84.07 | 51.76 | 95.31 |
| Fe500-5 | 65224 | 61283 | 25622688 | 418 | 84.41 | 51.53 | 93.96 |
| Fe500-6 | 60952 | 58410 | 24283982 | 415 | 86 | 51.93 | 95.83 |
| Fe800-1 | 72385 | 67232 | 28554590 | 424 | 83.49 | 51.05 | 92.88 |
| Fe800-2 | 70220 | 67843 | 28496243 | 420 | 85.72 | 51.71 | 96.61 |
| Fe800-3 | 70650 | 64610 | 27441246 | 424 | 86.84 | 50.87 | 91.45 |
| Fe800-4 | 55329 | 51661 | 21854267 | 423 | 86.85 | 51.04 | 93.37 |
| Fe800-5 | 82050 | 80150 | 34138584 | 425 | 85.17 | 50.74 | 97.68 |
| Fe800-6 | 78248 | 75075 | 31712523 | 422 | 82.03 | 52.11 | 95.94 |
| average | 71905 | 67391 | 28241444 | 418 | 84.5 | 51.99 | 93.63 |

Raw_reads(#), Clean_Reads(#), Base(nt), AvgLen(nt), Q20, GC%, and Effective% of each sample in Fe50 (n=8), Fe100 (n=8), Fe200(n=8), Fe500 (n=6), and Fe800 (n=6) groups.

**Supplementary Table 3 Alpha diversity indices of the cecal microbiota of piglets**

|  | **Fe50** | **Fe500** | **Fe800** | **SEM** | ***P*-value** |
| --- | --- | --- | --- | --- | --- |
| Shannon | 4.252a | 3.458a | 2.804b | 0.295 | 0.0011 |
| Simpson | 0.834a | 0.734ab | 0.636b | 0.048 | 0.0077 |
| Chao1 | 372.258a | 355.007a | 227.019b | 29.787 | 0.0013 |
| ACE | 385.354a | 367.13a | 234.53b | 30.645 | 0.001 |

Richness estimator (Chao1 and ACE), and diversity estimator (Shannon and Simpson) were analyzed by mothur software of the piglets from Fe50 (n=8), Fe500 (n=7), and Fe800 (n=6) groups. Ordinary one-way ANOVA or Kruskal-Wallis test was used for the statistical analysis among different groups. Data were presented as Mean ± SEM. Means in a row that have no superscript shared are significantly different from each other (P<0.05).

**Supplementary Table 4 The top 10 taxa at the phylum, class, order, family, genus level of cecal microbiota**

| **Taxonomy** | **Fe50** | **Fe500** | **Fe800** | **SEM** | ***P*-value** |
| --- | --- | --- | --- | --- | --- |
| **Phylum** |  |  |  |  |  |
| Firmicutes | 0.9227 | 0.9338 | 0.9512 | 0.0263 | 0.688 |
| Bacteroidetes | 0.0153 | 0.0394 | 0.0052 | 0.0022 | 0.0612 |
| Proteobacteria | 0.0336 | 0.0080 | 0.0339 | 0.0251 | 0.7519 |
| Spirochaetes | 0.0133^a^ | 0.0024^ab^ | 0.0009^c^ | 0.0008 | 0.3494 |
| Actinobacteria | 0.0058 | 0.0067 | 0.0069 | 0.0002 | 0.6959 |
| Tenericutes | 0.0058^a^ | 0.0061^ab^ | 0.0013^b^ | 0.0004 | 0.015 |
| Cyanobacteria | 0.0013 | 0.0005 | 0.0005 | 0.0004 | 0.6861 |
| Saccharibacteria | 0.0011^a^ | 0.0028^ab^ | 0.0001^b^ | 0.0000 | 0.0186 |
| Verrucomicrobia | 0.0006 | 0.0000 | 0.0000 | 0.0000 | 0.3118 |
| Chlamydiae | 0.0002 | 0.0001 | 0.0000 | 0.0000 | 0.7078 |
| Others | 0.0002 | 0.0002 | 0.0000 | 0.0000 | 0.1003 |
| **Class** |  |  |  |  |  |
| Bacilli | 0.5784^c^ | 0.6185^b^ | 0.7665^a^ | 0.0309 | 0.0301 |
| Clostridia | 0.3155^b^ | 0.2931^b^ | 0.1738^a^ | 0.0250 | 0.0178 |
| Erysipelotrichia | 0.0276 | 0.0210 | 0.0103 | 0.0040 | 0.6308 |
| Bacteroidia | 0.0153 | 0.0393 | 0.0052 | 0.0022 | 0.0604 |
| Gammaproteobacteria | 0.0095 | 0.0074 | 0.0307 | 0.0256 | 0.4017 |
| Epsilonproteobacteria | 0.0240 | 0.0002 | 0.0012 | 0.0013 | 0.1092 |
| unidentified_Spirochaetes | 0.0133 | 0.0024 | 0.0009 | 0.0008 | 0.3494 |
| Mollicutes | 0.0058 | 0.0061 | 0.0013 | 0.0004 | 0.855 |
| unidentified_Actinobacteria | 0.0004 | 0.0026 | 0.0056 | 0.0001 | 0.1518 |
| Coriobacteriia | 0.0055 | 0.0041 | 0.0012 | 0.0003 | 0.8014 |
| Others | 0.0046 | 0.0052 | 0.0032 | 0.0021 | 0.5737 |
| **Order** |  |  |  |  |  |
| Lactobacillales | 0.5784^c^ | 0.6184^b^ | 0.7624^a^ | 0.0308 | 0.0384 |
| Clostridiales | 0.3154^b^ | 0.2931^b^ | 0.1738^a^ | 0.0250 | 0.0178 |
| Erysipelotrichales | 0.0276 | 0.0210 | 0.0103 | 0.0040 | 0.6308 |
| Bacteroidales | 0.0153 | 0.0393 | 0.0052 | 0.0022 | 0.0606 |
| Enterobacteriales | 0.0019 | 0.0022 | 0.0256 | 0.0260 | 0.3887 |
| Campylobacterales | 0.0240 | 0.0002 | 0.0012 | 0.0013 | 0.1092 |
| Spirochaetales | 0.0133 | 0.0024 | 0.0009 | 0.0008 | 0.3494 |
| Mollicutes_RF9 | 0.0057^a^ | 0.0060^ab^ | 0.0013^b^ | 0.0004 | 0.0253 |
| Coriobacteriales | 0.0055 | 0.0041 | 0.0012 | 0.0003 | 0.4649 |
| Pasteurellales | 0.0058 | 0.0047 | 0.0048 | 0.0050 | 0.8866 |
| Others | 0.0071 | 0.0084 | 0.0132 | 0.0021 | 0.5673 |
| **Family** |  |  |  |  |  |
| Lactobacillaceae | 0.5723^c^ | 0.6178^b^ | 0.7409^a^ | 0.0308 | 0.0343 |
| Clostridiaceae_1 | 0.1182^b^ | 0.0219^ab^ | 0.0918^b^ | 0.0023 | 0.0349 |
| Ruminococcaceae | 0.1173 | 0.2063 | 0.0658 | 0.0215 | 0.1326 |
| Erysipelotrichaceae | 0.0276 | 0.0210 | 0.0103 | 0.0040 | 0.5442 |
| Peptostreptococcaceae | 0.0289 | 0.0080 | 0.0007 | 0.0003 | 0.5439 |
| Lachnospiraceae | 0.0420 | 0.0470 | 0.0136 | 0.0048 | 0.1257 |
| Enterobacteriaceae | 0.0019 | 0.0022 | 0.0256 | 0.0260 | 0.3887 |
| Christensenellaceae | 0.0069 | 0.0069 | 0.0013 | 0.0006 | 0.2817 |
| Bacteroidales_S24-7_group | 0.0040 | 0.0194 | 0.0020 | 0.0010 | 0.4340 |
| Streptococcaceae | 0.0058 | 0.0005 | 0.0183 | 0.0003 | 0.3186 |
| Others | 0.0749 | 0.0489 | 0.0298 | 0.0057 | 0.1646 |
| **Genus** |  |  |  |  |  |
| Lactobacillus | 0.5723^c^ | 0.6178^b^ | 0.7409^a^ | 0.0308 | 0.0343 |
| Clostridium_sensu_stricto_1 | 0.1161^a^ | 0.021a^b^ | 0.0913^b^ | 0.0022 | 0.4381 |
| Subdoligranulum | 0.0237 | 0.0377 | 0.0087 | 0.0026 | 0.0729 |
| Romboutsia | 0.0258 | 0.0067 | 0.0005 | 0.0003 | 0.5553 |
| Faecalibacterium | 0.0062 | 0.0340 | 0.0068 | 0.0054 | 0.2912 |
| Ruminococcaceae_UCG-014 | 0.0275 | 0.0551 | 0.0280 | 0.0145 | 0.5886 |
| Lachnospiraceae_XPB1014_group | 0.0089 | 0.0104 | 0.0018 | 0.0011 | 0.0929 |
| Escherichia-Shigella | 0.0019 | 0.0021 | 0.0237 | 0.0260 | 0.3893 |
| Ruminococcaceae_UCG-005 | 0.0210 | 0.0195 | 0.0067 | 0.0068 | 0.4120 |
| Streptococcus | 0.0058 | 0.0005 | 0.0182 | 0.0003 | 0.3179 |

Top 10 microbial population at different taxonomy level in cecum of piglets from Fe50 (n=8), Fe500 (n=7), and Fe800 (n=6) groups. Ordinary one-way ANOVA or Kruskal-Wallis test was used for the statistical analysis among different groups. Data were presented as Mean ± SEM. Means in a row that have no superscript shared are significantly different from each other (P<0.05).

**Supplementary Table 5 Data pre-processing statistics and quality control of RNA-sequencing**

| **sample** | **raw_**  **reads** | **clean_**  **reads** | **clean_**  **bases** | **error_**  **rate** | **Q20** | **Q30** | **GC_pct** | **GC_pct** | **rRNA_pct** |
| --- | --- | --- | --- | --- | --- | --- | --- | --- | --- |
| Fe50-1 | 27390344 | 26761684 | 8.03G | 0.03 | 97.1 | 92.43 | 54.71 | 54.71 | 0.63 |
| Fe50-3 | 28345630 | 27717076 | 8.32G | 0.03 | 96.82 | 91.79 | 54.1 | 54.1 | 0.6 |
| Fe50-4 | 30185622 | 29745789 | 8.92G | 0.03 | 97.07 | 92.27 | 52.65 | 52.65 | 0.58 |
| Fe50-5 | 27919292 | 27511946 | 8.25G | 0.03 | 96.8 | 91.71 | 54.47 | 54.47 | 0.36 |
| Fe50-6 | 28262668 | 27680822 | 8.3G | 0.03 | 97.04 | 92.26 | 54.03 | 54.03 | 0.4 |
| Fe800-1 | 35312260 | 34544148 | 10.36G | 0.03 | 96.31 | 94.63 | 54.55 | 54.55 | 0.43 |
| Fe800-2 | 28222247 | 27854915 | 8.36G | 0.03 | 96.23 | 94.53 | 54.77 | 54.77 | 0.44 |
| Fe800-6 | 29026010 | 28279548 | 8.48G | 0.03 | 96.72 | 95.19 | 53 | 53 | 0.42 |
| Fe800-3 | 30934019 | 30303068 | 9.09G | 0.03 | 96.45 | 91.04 | 53.91 | 53.91 | 0.36 |
| Fe800-4 | 30591449 | 30055898 | 9.02G | 0.03 | 96.67 | 95.13 | 54.17 | 54.17 | 0.41 |
| average | 29618954 | 29045489 | 8.71G | 0.03 | 96.72 | 93.10 | 54.04 | 54.04 | 0.463 |

raw_reads, clean_reads, clean_bases, error_rate, Q20, Q30, GC_pct, GC_pct, and rRNA_pct of each sample in Fe50 (n=5) and Fe800 (n=5) groups.
